# The role of allogeneic platelet-rich plasma in patients with diabetic foot ulcer: Current perspectives and future challenges

**DOI:** 10.3389/fbioe.2022.993436

**Published:** 2022-09-29

**Authors:** Min He, Tianyi Chen, Yuhuan Lv, Peiyang Song, Bo Deng, Xuewen Guo, Shunli Rui, Johnson Boey, David G. Armstrong, Yu Ma, Wuquan Deng

**Affiliations:** ^1^ Department of Endocrinology, Chongqing Emergency Medical Center, Chongqing University Central Hospital, School of Medicine, Chongqing University, Chongqing, China; ^2^ General Practice Department, Chongqing Southwest Hospital, Chongqing, China; ^3^ Department of Podiatry, National University Hospital, Singapore, Singapore; ^4^ Department of Surgery, Keck School of Medicine of University of Southern California, Los Angeles, CA, United States

**Keywords:** allogeneic platelet-rich plasma, wound healing, current perspectives, future challenges, diabetic foot ulcer

## Abstract

The frequency of chronic cutaneous wounds are sharply increasing in aging populations. Patients with age-related diseases, such as diabetes, tumors, renal failure and stroke are prone to soft tissue and skin injury, compounded by slowed healing in aging. Imbalance of wound inflammation, loss of growth factor secretion, and impairment of tissue repair abilities are all possible reasons for failed healing. Therefore, it is vital to explore novel approaches to accelerate wound healing. Platelet-rich plasma (PRP) as a cell therapy has been widely applied for tissue repair and regeneration. PRP promotes wound healing by releasing antimicrobial peptides, growth factors and micro-RNAs. Medical evidence indicates that autologous platelet-rich plasma (au-PRP) can promote wound healing effectively, safely and rapidly. However, its clinical application is usually restricted to patients with chronic cutaneous wounds, generally because of other severe complications and poor clinical comorbidities. Allogeneic platelet-rich plasma (al-PRP), with abundant sources, has demonstrated its superiority in the field of chronic wound treatment. Al-PRP could overcome the limitations of au-PRP and has promising prospects in clinical applications. The aim of this review is to summarize the current status and future challenges of al-PRP in chronic cutaneous wound management. We also summarized clinical cases to further describe the application of al-PRP for chronic wounds in clinical practice.

## Introduction

In recent decades, the frequency of chronic cutaneous wounds has significantly increased, likely due to health issues in the aging population (Jiang et al., 2011; Graves et al., 2022). Patients with diabetes mellitus (DM), tumors and surgeries can experience soft tissue injury because of long-term bed rest and mobility problems (Armstrong et al., 2017; Xu et al., 2022). Diabetic foot ulcers (DFU), pressure ulcers, venous ulcers and arterial insufficiency ulcers greatly lower quality of life, longevity, and impact financial and social burdens (Xu and Ran, 2016; Kapp and Santamaria, 2017; Graves et al., 2022). An epidemiological study of chronic wounds revealed that its prevalence in Wales was 6% in 2012–13 (Phillips et al., 2016). Chronic cutaneous wounds have different causative etiologies (Jiang et al., 2022). DFU is one of most common chronic diabetes-related injuries and has become the leading cause of nontramatic amputation and all-cause mortality, due to the high prevalence of DM (Xie et al., 2022). Here, we mainly focused on exploring a novel approach for chronic cutaneous wound healing, especially for patients with DFU.

DFU is one of most severe complications in patients with DM. The incidence of DFU is approximately 6.3% (Zhang et al., 2017). DFU wounds are difficult to heal, and the 5-year mortality after amputation reaches 40% (Wang et al., 2014; Phillips et al., 2016; Xu and Ran, 2016). It has been reported that DFU may have a worse prognosis than some forms of cancer (Armstrong et al., 2020). Patients with DFU always have a poor systemic condition, poor skin wound-healing performance and ulcer recurrence (Jiang et al., 2021). Hence, exploring a new, prompt, effective and safe approach for the promotion of DFU closure is key to reduce morbidity and mortality.

### The current treatment for diabetic foot ulcers

The main reasons for the difficulty associated with DFU healing are infection, impaired tissue repair function, and loss of growth factor secretion (Pietramaggiori et al., 2008; Meyer et al., 2011; Deng et al., 2021). There are 22 clinical practice guidelines for diabetic foot. Based on the recommendations, current standard of care for DFU care mainly involves offloading of pressure, wound care, choice of shoes and adjunctive treatment. Among these recommendations, promotion of DFU healing is extremely important for prevention of ulcer and its recurrence (Sun et al., 2022). Except for wound debridement and vascular intervention treatment, cell therapy is also a promising approach for DFU, including stem cell, platelet-rich plasma (PRP) and its derived growth factors. After activation by thrombin and calcium, PRP released transforming growth factor, platelet-derived growth factor, insulin-like growth factor, fibroblast growth factor and vascular endothelial growth factor, which play an important role in tissue repair and regeneration (Carter et al., 2003). In 1998, Marx et al. demonstrated novel data on the effectiveness of PRP in the treatment of bone defects, which attracted the attention of the wound repair and regeneration fields (Marx et al., 1998). Later, researchers found that PRP can accelerate wound healing and serve as an antibiosis agent (Bielecki et al., 2007; Li and Li, 2013; López et al., 2014; Li et al., 2019a). PRP is regarded as a reservoir of host defense factors, and these secreted factors assist the host against the bacteria (Tamagawa-Mineoka, 2015). Both functions have also been proven in animal experiments (Maciel et al., 2012). Moreover, PRP can reduce the recurrence rate, infection rate, amputation rate, mortality and medical costs in the treatment of DFU (Dougherty, 2008). PRP can be harvested from autologous or allogeneic whole blood. Autologous platelet-rich plasma (au-PRP) has already been successfully applied in the treatment of chronic refractory wounds for many years. In our previous reports, diabetic patients with acute necrotizing fasciitis and refractory diabetic foot benefited from treatment with au-PRP (Deng et al., 2016; Jiang et al., 2020). Furthermore, evidence-based medical studies also supported the notion that au-PRP could improve the healing of chronic wounds, especially for DFU (Martinez-Zapata et al., 2016). However, diabetic patients with severe acute or chronic complications or coexisting diseases, such as peripheral arterial and neuropathic disease (Zhang et al., 2022), thrombosis, anemia, thrombocytopenia, or malnutrition are usually restricted from the utilization of au-PRP. Alternatively, allogeneic platelet-rich plasma (al-PRP) has already been identified to promote the healing of chronic wounds in our and other previous studies (Villela and Santos, 2010; Semenič et al., 2018; Liao et al., 2020). PRP can be harvested from patients as autologous preparations. However, under diabetic condition, poor platelet number, reduced cell activity or impaired PRP efficacy may limit their use. Administration of allogeneic PRP donated by healthy and/or younger individuals is regarded with increasing interest to overcome such limitation (He et al., 2020). Al-PRP may overcome the shortcomings of au-PRP and provide a new strategy in adjunct treatment of chronic cutaneous wounds. The aim of this review is to summarize the current and prospective challenges of PRP treatments, as well as review key case that used al-PRP in the management of chronic cutaneous wounds.

### Preparation protocol for platelet-rich plasma

Platelet-rich plasma (PRP) contains high concentrations of the growth factors (Tian et al., 2019), antibacterial agent (Li et al., 2019a; Sethi et al., 2021). Based on recently reports of systematic review and meta-analysis, current evidence suggests that autologous PRP may increase wound closure, shorten healing time in patients with DFU (Li et al., 2019b; Dai et al., 2020; Qu et al., 2021), but both these studies and the International Working Group of the Diabetic Foot (IWGDF) working group indicate that a good design and of a high quality are needed before widely application (Game et al., 2016).

The inconsistent clinical effect of PRP for DFU, the preparation protocol may be considered as main cause. Numerous studies have demonstrated the preparation protocol of PRP by separating whole blood *via* gradient centrifugal stratification. However, as mentioned in a recent systematic review study, the composition of PRP was completely reported less than 30% in enrolled forty-one studies. Two ranges could be identified for platelet concentration, the first between 0.14 × 10^6^ and 0.80 × 10^6^ platelets/µl and the second between 1.086 × 10^6^ and 10 ×10^6^ platelets/µl. That is to say, efficacy of PRP depends greatly on its preparation approach. Therefore, there is a need to standardized methodology of PRP preparation as well as its guidelines creation (Anitua et al., 2022; Mastrogiacomo et al., 2022).

### Au-PRP applications and limitations in diabetic foot ulcers

Au-PRP is extracted by centrifugation and separation from peripheral whole blood, and then a gel is formulated by activation with a mixed solution of thrombin and calcium. Recently, we published a report on a severely infective diabetic patient with foot necrotising fasciitis and gaseous gangrene, who successfully recovered and underwent limb salvage after a combination of clinical interventions that included PRP (Deng et al., 2016). We recently also reported another case with refractory chronic DFU in which amputation was successfully prevented through a combination approach of negative pressure wound therapy, au-PRP and antibiotic bone-cement (Jiang et al., 2020). Furthermore, a study by Babaei et al. (2017) recruited 150 patients with DFUs treated with au-PRP, and showed that DFUs with a wound size of 2–2.5 cm^2^ completely healed after 7.2 weeks, a wound size of 5.8–8.5 cm^2^ healed after 7.5 weeks, and a wound size of 8.5–12.5 cm^2^ healed after 8.8 weeks. Interestingly, there was no recurrence after an 8-month follow-up. Au-PRP could promote wound healing more quickly and effectively than traditional antibiotic dressings (Deng et al., 2016; Ahmed et al., 2017; Babaei et al., 2017; Jiang et al., 2020). Based on a literature review, PRP as a cell therapy could not only accelerate wound closure but also improve wound infection as an adjunct antibacterial agent. In addition, in our previous empirical study, PRP played an important role in anti-inflammation, anti-infection and promotion of wound healing (Li et al., 2019a).

Au-PRP is now widely used in tissue repair and regeneration in medical cosmetology (Lee et al., 2019), ophthalmology (Avila et al., 2018), orthopedics (McCarrel et al., 2014), neurology (Ye et al., 2012), and oral and maxillofacial surgery (Anitua, 1999; Piccin et al., 2016). Wounds and incisions, however, are usually in an acute state, even though most patients with chronic refractory wounds have experienced a long history of chronic disease (Yotsu et al., 2015). These chronic disease states are often accompanied by acquired infection, kidney disease, vascular disease and tumors. Some of these patients may also experience malnutrition, frailty, gastrointestinal hemorrhage, anemia and cachexia. All these factors make it difficult to prepare au-PRP and ultimately restrict its applicability. Hence, based on our experience and research reports, we suggest that au-PRP treatment should not be recommended if a patient is experiencing one of the following comorbidities: current or recent chemotherapy treatment for cancer; chronic diseases such as severe high blood pressure, acute coronary disease, severe renal dysfunction, or gastrointestinal hemorrhage; hematological diseases such as anemia, thrombocytopenia, leukemia, or lymphoma; cachexia; or immune deficiency. Therefore, al-PRP may be considered an alternative to PRP for chronic wound treatment.

### The methodology of al-PRP preparation

**FIGURE 1:**
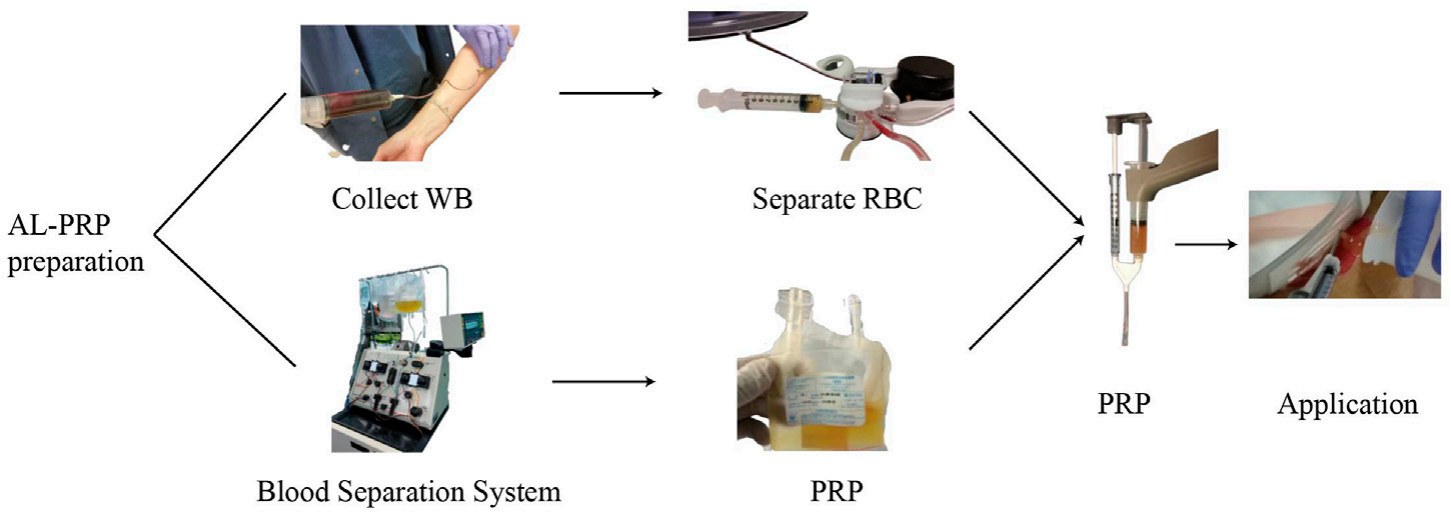
A schematic diagram of allogenic PRP treatment. There are two different preparation methodology of allogenic PRP donated from healthy volunteers: manual platelet separation with venous whole blood or platelets apheresis with automatic machine only collected platelets. The allogenic platelet rich gel was used to diabetic foot ulcer after activation by with a mixed solution of thrombin and calcium.

Al-PRP is extracted from banked or fresh platelets donated from healthy subjects. The preparation methods of al-PRP mainly including: manual platelet separation with venous whole blood or platelets apheresis with automatic machine only collected platelets (Figure 1). The preparation of al-PRP by the centrifugal separation method has lower requirements for preparation devices, is cost effective, and the actual procedural steps are simple and feasible. It is the most widely used preparation method at present. An increasing number of studies have identified the efficacy and safety of al-PRP for chronic wounds. Thus, the application of al-PRP has received more attention in the field of chronic wound repair, but patients and physicians worry about its safety in clinical practice (Akbarzadeh et al., 2021). We have carefully reviewed the literature through December 2021 to discuss its efficacy and safety (Table 1).

**TABLE 1:** Summary of studies on efficacy and safety of al-PRP for wound healing.

Author Year	Study design	Participants/Wound	Intervention	Outcomes/Adverse effects
He et al. (2020)	Observational cohort study	75 patients with DLEUs al-PRP (*n* = 20) au-PRP (*n* = 25) SWT (*n* = 30)	Source: PRP from banked blood Centrifuge 1: 600 g for 15 min Centrifuge 2: 1,135 g for 7 min al-PRP Concentration: mean (1,043 ± 180.3) ×10^9^/L au-PRP Concentration: mean (939 ± 237.4) ×10^9^/L Treatment Regime 1. Standard care 2. After wound debridement with al-PRP or au-PRP	The wound healing times of the al-PRP group (56.9 ± 29.22 days) and au-PRP group (55.6 ± 33.8 days) were significantly shorter than those of the CWT group (88.0 ± 43.4 days) (*p* < 0.05) No obvious adverse reactions (fever, edema, pain, skin itching, rash, or other sensory abnormalities)
Liao et al. (2020)	Randomized Controlled trial	60 patients with refractory wounds	Source: PRP from family member donation Centrifuge 1: 400 g for 10 min Centrifuge 2: 1,200 g for 20 min al-PRP Concentration: 1,200 ×10^9^/L Treatment Regime 1. Wound debridement + saline dressing 2. Dressed in PRP	Significant improved rate of healing at week 1 and week 3 time points (*p* < 0.05) Control: conventional skin grafting No adverse reactions
Semenič et al. (2018)	Randomized Controlled trial	60 patients with ulcers of different aetiologies	Source: ABO matched donation Centrifuge: not reported PRP Concentration: not reported Treatment Regime: 1. Wound irrigated with antiseptic 2. PRP gel applied, covered with dressing 3.A total of treatment for 3 times	Wound size decreased to 35.01% compared to 89.95% in controls (*p* < 0.001) at 6 months No adverse reactions observed
Asadis et al. (2014)	Case series	10 patients with refractory ulcer failed to healing by traditional therapies	Source: ABO matched peripheral blood Centrifuge 1: 2,000 g for 2 min Centrifuge 2: 4,000 g for 8 min PRP Concentration: not reported Treatment Regime: sacrum wound	9 patients completely healed, 1 patient with the area of wound reduced significantly
Scevola et al. (2010)	Randomized Controlled trial	13 patients with pressure sores	Source: platelet concentrate from hospital Centrifuge: not reported PRP Concentration: more than 2.0×10^10^ platelets transplanted Treatment Regime: 1. Clean wound bed 2. Twice weekly for 18 weeks Control: no platelet gel applied	Improved granulation tissue proliferation in first 2 weeks No changes in ulcer bacteria contents, nor serum signs of infection
Jeong et al. (2010)	Randomized Controlled trial	100 patients with diabetic foot ulcers	Source: blood bank platelet concentrate Centrifuge: 3,000 g for 30 min PRP Concentration: mean 1.1×10^10^ ± 3.9×10^9^ platelets transplanted Treatment Regime: 1. Wounds debridement 2. Topical application of 12.5 or 25 ml of concentrate plus fibrinogen, 2 doses 3–4 days apart Control: topical fibrinogen and thrombin	79% complete healing at 12 weeks Mean healing time 7.0 ± 1.9 weeks, compared to 9.1 ± 2.2 weeks in controls No adverse effects in each of the groups

DLEUs, diabetic lower extremity ulcers; al-PRP, allogeneic platelet-rich plasma; au-PRP, autologous platelet-rich plasma; CWT, conventional wound therapy; PRP, platelet-rich plasma.

### Al-PRP application for chronic wound repair

In 2007, Smrke et al. (2007) performed a combined therapy for delayed healing of severe fracture wounds using autologous cancellous bone transplantation and al-PRP. The fracture wound healed after 6 months, the patient could walk on the healed leg, and both legs were of the same length. In 2010, Scevola et al. (2010) recruited 13 patients who suffered pressure sores because of prolonged bed rest secondary to spinal injuries. Even though there was no significant difference in the final reduced ulcer area, al-PRP gel was more effective in accelerating ulcer healing in the first 2 weeks than treatment with the best dressing. In 2013, Tashnizi et al. (2013) recruited six patients with life-threatening, chronic sternal wounds and multidrug-resistant septicemia. After a mixture of al-PRP and fibrin application, five cases completely healed, and one case had a reduced wound area. In 2014, ten patients with refractory ulcers that failed to heal by traditional therapies were treated with al-PRP; nine of the patients completely healed, and one patient had a significantly reduced wound area (Asadis et al., 2014). In 2015, Costanzo et al. (2015) used al-PRP in a multiple myeloma patient who suffered skin flap necrosis because of the exudative lesion on the left hand caused by the leakage of chemical medicine. The wound completely healed after 12 al-PRP treatments, and no recurrence was observed at the 19-month follow-up. There were no adverse events reported in the above clinical studies.

In recent case reports, al-PRP was proven to be safe and efficacious for chronic wounds either as an application of al-PRP alone (Oliveira et al., 2017; Zhao et al., 2019) or in combination as an adjuvant method (Hernandez et al., 2012; Ju et al., 2021). Furthermore, a prospective, randomized, single-center study was conducted recently and enrolled 60 patients with chronic wounds. The wounds in the al-PRP group were treated by standard care and al-PRP, while the control group was treated only by standard care. The combined treatment of chronic wounds by standard care and al-PRP significantly shortened healing time, suggesting that al-PRP is an effective, safe, adjuvant treatment for chronic wounds (Liao et al., 2020).

**FIGURE 2:**
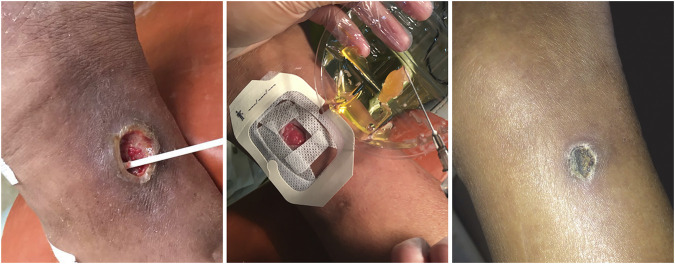
The diabetic lower extremity ulcer successfully healed with allogenic PRP. The patient with diabetic lower extremity ulcer was topical administrated with allogenic PRP. After 21-day treatment, the ulcer was completely healed.

Based on our long-term research on DFUs, we also performed an observational study to compare the efficacy and safety of al-PRP and au-PRP for diabetic lower extremity ulcers, one of patient with DFU was successfully healed by al-PRP (Figure 2). Seventy-five patients with diabetic lower extremity ulcers were divided into three groups according to age and ulcer size. We found that the wound healing times in the al-PRP group and au-PRP group were significantly shorter than those in the standard care group. Although there was no significant difference in the daily healing area among all groups, the trend of the healing rate in the al-PRP group, au-PRP group, and standard care group gradually decreased. No obvious adverse reactions (fever, edema, pain, skin itching, rash, or other sensory abnormalities) were observed in either the au-PRP or the al-PRP groups. Al-PRP could effectively and safely promote wound healing in patients with diabetic lower extremity ulcers as an off-the-shelf solution when au-PRP is not indicated (He et al., 2020).

### Basic research trends of al-PRP in wound treatment

The promotion of wound healing depends on the amount of platelet content and whether platelet function is intact. Studies have found that the platelet turnover rate of diabetic mice is increased (Anitua, 1999), and the content of growth factors and proteins that promote ulcer healing in the autologous plasma of diabetic animals are decreased (Tyndall et al., 2003). In addition, platelet function is also related to patient age. With age, oxidative stress increases correspondingly, and the ability of nitric oxide to inhibit platelet activation is affected (Chris, 2016). One study demonstrated that platelet aggregation, integrin αIIbβ3 and P-selectin exposure were negatively correlated with age (Gilstad et al., 2009). The content and expression of platelet microRNAs (miRNAs) were also affected by age and activation approach (Simon et al., 2014; Rui et al., 2021). Al-PRP is derived from healthy adult blood donors, is less affected by the above factors and has a more stable curative effect.

In 2015, Tae-ho et al. (2015) used al-PRP to treat beagle dogs with acute pancreatitis complicated by disseminated intravascular coagulation causing large-area skin defects. After 2 days of treatment, granulation tissue began to grow. After 21 days, the wounds were completely healed. Another study confirmed that intra-articular injection of al-PRP combined with water activity can effectively promote the repair of tendon injury in rats (Rajabi et al., 2015). In 2017, Abouelnasr et al. (2017) confirmed through dog experiments that al-PRP can repair abdominal wall defects and reduce peritoneal adhesions and recurrence rates by promoting neovascularization and increasing tissue deposition and fusion. In 2022, Saputro et al. (2022) confirmed that the application of allogenic freeze-dried PRP was able to increase the number of fibroblasts and neovascularization in the full-thickness wound-healing process in rabbits. The allogenic freeze-dried platelet-rich plasma increased the number of fibroblasts and neovascularization in wound healing. Allogeneic platelet concentrates derived from healthy blood donors have beneficial effects on various aspects of wound healing *in vitro* and are superior to plasma or platelets alone (van der Bijl et al., 2019). Al-PRP could be a promising bioactive scaffold due to its synergistic effect in supporting cell proliferation, maintaining cell viability and favoring extracellular matrix production; thus, al-PRP can likely be used as a biological scaffold for the delivery of chondroprogenitors in cartilage healing (Vinod et al., 2019).

### The immunological aspects of the application of al-PRP

Platelet-associated immunomodulatory molecules are mainly stored in platelet granules. Because of extremely high platelet numbers, amount of immune mediators are released to interact with various cells of the immune system. Platelet could express molecules and cytokines with immune functions including platelet-derived growth factor (PDGF)、transforming growth factor-β(TGFβ)、clusters of differentiation (CD) 40、CD154 and CXCL chemokine family to regulate different types of tissue damage (Semple et al., 2011). Furthermore, the growth factors and pro-inflammatory chemokines released by microvesicles are able to modulate the function of immune cells in type 2 diabetes (Cortez-Espinosa et al., 2017). Platelets are deeply involved in the innate and adaptive immune responses in the skin *via* interactions with leukocytes and the endothelium (Tamagawa-Mineoka, 2015).

At present, whether al-PRP will produce a severe immune response in patients is an important issue that has been widely studied by scholars worldwide. As Table 1 described, all reported clinical trials have demonstrated no serious allergic reactions to date. Most of clinical studies have indicated that al-PRP was topically applied to wound care with ABO matched donation. In an interesting *vitro* study, whether the differentiation of peripheral blood monocytes to dendritic cells *via* stimulation with granulocyte monocyte colony stimulating factor and interleukin-4 affected by PRP was investigated in order to define al-PRP mismatched for ABO and Rh antigens before clinical practice. The results suggested that al-PRP can promote the differentiation of monocytes to a regulatory anti-inflammatory population, possibly favouring wound healing (Papait et al., 2018). From animal experiments, it was also found that after intramuscular injection of allogeneic non-homologous PRP into rabbits, the number and proportion of CD4^+^ and CD8^+^ cells in the peripheral blood of the rabbits only increased slightly. Compared with before injection, the difference was not statistically significant, and there was no significant change in the histomorphology at the injection site (Zhang et al., 2013). Actually, the immunogenicity of al-PRP is negligible for the following reasons: First, al-PRP for wound healing is a topical product and does not enter the circulatory system. The human leukocyte antigen and human platelet antigen in the gel have limited contact with host antibodies. Second, after activation, the surface antigen structure and expression level of platelets are changed, and immunogenicity is reduced. Third, PRP can be completely degraded and absorbed within a few weeks, thus eliminating the possibility of a chronic immune response.

Al-PRP could easily obtain from healthy donator and solve the risk of poor standardization procedures in preparation of PRP. So far, homologous al-PRP has not been reported due to side effect problem. Hence, immunogenicity would not be a main obstacle to the application of al-PRP. Immunological aspect to topical application of al-PRP is little paid attention, further study is needed to identify its’ immune role in patients with diabetes or elderly.

In a meta-analysis study, the results indicate that al-PRP can significantly accelerate DFU healing compared with control, and no adverse events have been observed in any al-PRP experimental results (Gong et al., 2022). However, there was not a large-scale, randomized, controlled trial in the treatment of chronic DFU with al-PRP for the meta-analysis (Du et al., 2021; Mastrogiacomo et al., 2022). Of course, the promotion of each new technology is both an opportunity and a challenge for the medical industry. At present, al-PRP has not been widely used in the clinic, and certain issues still need to be further addressed. Further research is needed to determine whether the growth factors in al-PRP will not be fully expressed due to the depletion of other unknown cofactors, as well as to determine if al-PRP quality will decrease with the prolongation of storage time. Future studies should also investigate whether the use of al-PRP to repair wounds can reduce the ulcer recurrence rate, and whether the combined use of al-PRP and surgical debridement and/or other noninvasive dressings can produce synergistic effects. In addition, exosomes derived from platelets has been successfully prepared in our previous study (Rui et al., 2021). Au- or al-PRP derived exosomes could be considered as a preferred choice for “off-the shelf” ideal bio-material dressing for DFU treatment. Finally, further studies are needed to identify the efficacy and safety of al-PRP in the treatment of chronic wounds with a large sample size of individuals.

## Conclusion

The amputation and mortality of patients with DFU have sharply increased since the COVID-19 pandemic (Jeong et al., 2010). It is especially key to promote wound healing with a novel approach for elderly patients with chronic wounds. Al-PRP could be large-scale and with standardized production because of its easy preparation and application. Al-PRP is derived from healthy adult blood donors, the quality and content of platelets are stable, and the curative effect is reliable (Papait et al., 2018). The use of Al-PRP also avoids the health burden associated with autologous blood collection for the patients, thereby overcoming the shortcomings of au-PRP in clinical settings. In sum, Al-PRP may provide a new option in the treatment of chronic refractory wounds.

## References

[B1] Abouelnasr K. Hamed M. Lashen S. El-Adl M. Eltaysh R. Tagawa M. (2017). Enhancement of abdominal wall defect repair using allogenic platelet-rich plasma with commercial polyester/cotton fabric (Damour) in a canine model. J. Vet. Med. Sci. 79 (7), 1301–1309. 10.1292/jvms.17-0139 28603214 PMC5559380

[B2] Ahmed M. Reffat S. Hassan A. Eskander F. (2017). Platelet rich plasma for the treatment of clean diabetic foot ulcers. Ann. Vasc. Surg. 38, 206–211. 10.1016/j.avsg.2016.04.023 27522981

[B3] Akbarzadeh S. McKenzie M. B. Rahman M. M. Cleland H. (2021). Allogeneic platelet-rich plasma: Is it safe and effective for wound repair? Eur. Surg. Res. 62 (1), 1–9. 10.1159/000514223 33621973

[B4] Anitua E. (1999). Plasma rich in growth factors: Preliminary results of use in the preparation of future sites for implants. Int. J. Oral Maxillofac. Implants 14, 529 10453668

[B5] Anitua E. Zalduendo M. Troya M. Alkhraisat M. H. Blanco-Antona L. A. (2022). Platelet-rich plasma as an alternative to xenogeneic sera in cell-based therapies: A need for standardization. Int. J. Mol. Sci. 23 (12), 6552. 10.3390/ijms23126552 35742995 PMC9223511

[B6] Armstrong D. G. Boulton A. J. M. Bus S. A. (2017). Diabetic foot ulcers and their recurrence. N. Engl. J. Med. Overseas. Ed. 376 (24), 2367–2375. 10.1056/nejmra1615439 28614678

[B7] Armstrong D. G. Swerdlow M. A. Armstrong A. A. Conte M. S. Padula W. V. Bus S. A. (2020). Five year mortality and direct costs of care for people with diabetic foot complications are comparable to cancer. J. Foot Ankle Res. 13 (1), 16. 10.1186/s13047-020-00383-2 32209136 PMC7092527

[B8] Asadis M. Alamdari D. H. Rahimi H. R. Aliakbarian M. Jangjoo A. Abdollahi A. (2014). Treatment of life-threatening wounds with a combination of allogenic platelet-rich plasma, fibrin glue and collagen matrix, and a literature review. Exp. Ther. Med. 8 (2), 423–429. 10.3892/etm.2014.1747 25009595 PMC4079439

[B9] Avila M. Y. Igua A. M. Mora A. M. (2018). Randomised, prospective clinical trial of platelet-rich plasma injection in the management of severe dry eye. Br. J. Ophthalmol. 103, 648–653. 10.1136/bjophthalmol-2018-312072 29970389

[B10] Babaei V. Afradi H. Gohardani H. Z. Nasseri F. Azarafza M. Teimourian S. (2017). Management of chronic diabetic foot ulcers using platelet-rich plasma. J. Wound Care 26 (12), 784–787. 10.12968/jowc.2017.26.12.784 29244965

[B11] Bielecki T. M. Gazdzik T. S. Arendt J. Szczepanski T. Krol W. Wielkoszynski T. (2007). Antibacterial effect of autologous platelet gel enriched with growth factors and other active substances: An *in vitro* study. J. Bone Jt. Surg. Br. volume 89 (3), 417–420. 10.1302/0301-620x.89b3.18491 17356164

[B12] Carter C. A. Jolly D. G. Worden C. E. Hendren D. G. Kane C. J. (2003). Platelet-rich plasma gel promotes differentiation and regeneration during equine wound healing. Exp. Mol. Pathol. 74, 244–255. 10.1016/s0014-4800(03)00017-0 12782011

[B13] Chris I. J. (2016). Platelet function and ageing. Mamm. Genome 27, 358–366. 10.1007/s00335-016-9629-8 27068925 PMC4935731

[B14] Cortez-Espinosa N. Mayoral L. P. Perez-Campos E. Alejandro Cabrera Fuentes H. Perez-Campos Mayoral E. Martinez-Cruz R. (2017). Platelets and platelet-derived microvesicles as immune effectors in type 2 diabetes. Curr. Vasc. Pharmacol. 15 (3), 207–217. 10.2174/1570161115666170126130309 28128064

[B15] Costanzo G. D. Loquercio G. Marcacci G. Iervolino V. Mori S. Petruzziello A. (2015). Use of allogeneic platelet gel in the management of chemotherapy extravasation injuries: A case report. Onco. Targets. Ther. 8, 401–404. 10.2147/ott.s68469 25709472 PMC4332310

[B16] Dai J. Jiang C. Sun Y. Chen H. (2020). Autologous platelet-rich plasma treatment for patients with diabetic foot ulcers: A meta-analysis of randomized studies. J. Diabetes Complicat. 34 (8), 107611. 10.1016/j.jdiacomp.2020.107611 32402839

[B17] Deng L. Du C. Song P. Chen T. Rui S. Armstrong D. G. (2021). The role of oxidative stress and antioxidants in diabetic wound healing. Oxid. Med. Cell. Longev. 2021, 1–11. 10.1155/2021/8852759 PMC7884160 33628388

[B18] Deng W. Boey J. Chen B. Byun S. Lew E. Liang Z. (2016). Platelet-rich plasma, bilayered acellular matrix grafting and negative pressure wound therapy in diabetic foot infection. J. Wound Care 25 (7), 393–397. 10.12968/jowc.2016.25.7.393 27410393

[B19] Dougherty E. J. (2008). An evidence-based model comparing the cost-effectiveness of platelet-rich plasma gel to alternative therapies for patients with nonhealing diabetic foot ulcers. Adv. Skin. Wound Care 21 (12), 568–575. 10.1097/01.asw.0000323589.27605.71 19065083

[B20] Du C. Li Y. Xie P. Zhang X. Deng B. Wang G. (2021). The amputation and mortality of inpatients with diabetic foot ulceration in the COVID-19 pandemic and postpandemic era: A machine learning study. Int. Wound J. Online ahead of print. 10.1111/iwj.13723 PMC9493239 34818691

[B21] Game F. L. Apelqvist J. Attinger C. Hartemann A. Hinchliffe R. J. Londahl M. (2016). Effectiveness of interventions to enhance healing of chronic ulcers of the foot in diabetes: A systematic review. Diabetes. Metab. Res. Rev. 32 (1), 154–168. 10.1002/dmrr.2707 26344936

[B22] Gilstad J. R. Gurbel P. A. Andersen R. E. (2009). Relationship between age and platelet activation in patients with stable and unstable angina. Arch. Gerontol. Geriatr. 48 (2), 155–159. 10.1016/j.archger.2007.12.006 18282622

[B23] Gong F. Zhang Y. Gao J. Li X. Zhang H. Ma G. (2022). Effect of platelet-rich plasma vs standard management for the treatment of diabetic foot ulcer wounds: A meta-analysis. Int. Wound J. 10.1111/iwj.13858 PMC9797932 35751432

[B24] Graves N. Phillips C. J. Harding K. (2022). A narrative review of the epidemiology and economics of chronic wounds. Br. J. Dermatol. 187 (2), 141–148. 10.1111/bjd.20692 34549421

[B25] He M. Guo X. Li T. Jiang X. Chen Y. Yuan Y. (2020). Comparison of allogeneic platelet-rich plasma with autologous platelet-rich plasma for the treatment of diabetic lower extremity ulcers. Cell. Transpl. 29, 096368972093142–096368972093149. 10.1177/0963689720931428 PMC7563928 32510240

[B26] Hernandez V. R. Vilahur G. Ferrer-Lorente R. Pena E. Badimon L. (2012). Platelets derived from the bone marrow of diabetic animals show dysregulated endoplasmic reticulum stress proteins that contribute to increased thrombosis. Arterioscler. Thromb. Vasc. Biol. 32 (9), 2141–2148. 10.1161/atvbaha.112.255281 22837468

[B27] Jeong S. H. Han S. K. Kim W. K. (2010). Treatment of diabetic foot ulcers using a blood bank platelet concentrate. Plastic Reconstr. Surg. 125 (3), 944–952. 10.1097/prs.0b013e3181cb6589 20195121

[B28] Jiang X. Deng F. Rui S. Ma Y. Wang M. Deng B. (2022). The evaluation of gait and balance for patients with early diabetic peripheral neuropathy: A cross-sectional study. Risk Manag. Healthc. Policy 15, 543–552. 10.2147/rmhp.s361698 35386278 PMC8977473

[B29] Jiang X. Li N. Yuan Y. Yang C. Chen Y. Ma Y. (2020). Limb salvage and prevention of ulcer recurrence in a chronic refractory diabetic foot osteomyelitis. Diabetes Metab. Syndr. Obes. 13, 2289–2296. 10.2147/dmso.s254586 32636663 PMC7335304

[B30] Jiang X. Yuan Y. Ma Y. Zhong M. Du C. Boey J. (2021). Pain management in people with diabetes-related chronic limb-threatening ischemia. J. Diabetes Res. 2021, 1–11. 10.1155/2021/6699292 PMC8128546 34046505

[B31] Jiang Y. Huang S. Fu X. Liu H. Ran X. Lu S. (2011). Epidemiology of chronic cutaneous wounds in China. Wound Repair Regen. 19 (2), 181–188. 10.1111/j.1524-475x.2010.00666.x 21362085

[B32] Ju W. Fu S. Huang J. Li B. Wang L. Zheng M. (2021). Using human umbilical cord mesenchymal stem cells combined with allogenic platelet-rich fibrin membrane for the treatment of dual limb ischemia in an elderly patient: A case report. Med. Baltim. 100 (10), e25068. 10.1097/md.0000000000025068 PMC7969242 33725897

[B33] Kapp S. Santamaria N. (2017). The financial and quality-of-life cost to patients living with a chronic wound in the community. Int. Wound J. 14 (6), 1108–1119. 10.1111/iwj.12767 28635188 PMC7949507

[B34] Lee Z. H. Sinno S. Poudrier G. Motosko C. C. Chiodo M. Saia W. (2019). Platelet rich plasma for photodamaged skin: A pilot study. J. Cosmet. Dermatol. 18 (1), 77–83. 10.1111/jocd.12676 29855132

[B35] Li H. Li B. (2013). PRP as a new approach to prevent infection: Preparation and *in vitro* antimicrobial properties of PRP. J. Vis. Exp. 74. 10.3791/50351 PMC3653398 23609458

[B36] Li T. Ma Y. Wang M. Wang T. Wei J. Ren R. (2019). Platelet-rich plasma plays an antibacterial, anti-inflammatory and cell proliferation-promoting role in an *in vitro* model for diabetic infected wounds. Infect. Drug Resist. 12, 297–309. 10.2147/idr.s186651 30774397 PMC6357877

[B37] Li Y. Gao Y. Gao Y. Chen D. Wang C. Liu G. (2019). Autologous platelet-rich gel treatment for diabetic chronic cutaneous ulcers: A meta-analysis of randomized controlled trials. J. Diabetes 11 (5), 359–369. 10.1111/1753-0407.12850 30182534

[B38] Liao X. Liang J. X. Li S. H. Huang S. Yan J. X. Xiao L. L. (2020). Allogeneic platelet-rich plasma therapy as an effective and safe adjuvant method for chronic wounds. J. Surg. Res. 246, 284–291. 10.1016/j.jss.2019.09.019 31622885

[B39] López C. Alvarez M. E. Carmona J. U. (2014). Temporal bacteriostatic effect and growth factor loss in equine platelet components and plasma cultured with methicillin-sensitive and methicillin-resistant staphylococcus aureus: A comparative *in vitro* study. Vet. Med. Int. 2014, 1–8. 10.1155/2014/525826 PMC4260436 25506468

[B40] Maciel F. B. DeRossi R. Módolo T. J. Pagliosa R. C. Leal C. R. Delben A. A. (2012). Scanning electron microscopy and microbiological evaluation of equine burn wound repair after platelet-rich plasma gel treatment. Burns 38 (7), 1058–1065. 10.1016/j.burns.2012.02.029 22683140

[B41] Martinez-Zapata M. J. Martí-Carvajal A. J. Solà I. Exposito J. A. Bolibar I. Rodriguez L. (2016). Autologous platelet-rich plasma for treating chronic wounds. Cochrane Database Syst. Rev. 5, CD006899. 10.1002/14651858.CD006899.pub3 PMC9308064 27223580

[B42] Marx R. E. Carlson E. R. Eichstaede R. M. Schimmele S. R. Strauss J. E. Georgeff K. R. (1998). Platelet-rich plasma: Growth factor enhancement for bone grafts. Oral Surg. Oral Med. Oral Pathol. Oral Radiol. Endod. 85, 638–646. 10.1016/s1079-2104(98)90029-4 9638695

[B43] Mastrogiacomo M. Nardini M. Collina M. C. Di Campli C. Filaci G. Cancedda R. (2022). Innovative cell and platelet rich plasma therapies for diabetic foot ulcer treatment: The allogeneic approach. Front. Bioeng. Biotechnol. 10, 869408. 10.3389/fbioe.2022.869408 35586557 PMC9108368

[B44] McCarrel T. M. Mall N. A. Lee A. S. Cole B. J. Butty D. C. Fortier L. A. (2014). Considerations for the use of platelet-rich plasma in orthopedics. Sports Med. 44, 1025–1036. 10.1007/s40279-014-0195-5 24760591

[B45] Meyer M. Müller A. K. Yang J. Sulcova J. Werner S. (2011). The role of chronic inflammation in cutaneous fibrosis: Fibroblast growth factor receptor deficiency in keratinocytes as an example. J. Investig. Dermatol. Symp. Proc. 15 (1), 48–52. 10.1038/jidsymp.2011.1 22076327

[B46] Oliveira M. G. Abbade L. P. Miot H. A. Ferreira R. R. Deffune E. (2017). Pilot study of homologous platelet gel in venous ulcers. An. Bras. Dermatol. 92 (4), 499–504. 10.1590/abd1806-4841.20175496 28954098 PMC5595596

[B47] Papait A. Cancedda R. Mastrogiacomo M. Poggi A. (2018). Allogeneic platelet-rich plasma affects monocyte differentiation to dendritic cells causing an anti-inflammatory microenvironment, putatively fostering wound healing. J. Tissue Eng. Regen. Med. 12 (1), 30–43. 10.1002/term.2361 27863082

[B48] Phillips C. J. Humphreys I. Fletcher J. Harding K. Chamberlain G. Macey S. (2016). Estimating the costs associated with the management of patients with chronic wounds using linked routine data. Int. Wound J. 13, 1193–1197. 10.1111/iwj.12443 25818405 PMC7949824

[B49] Piccin A. Di Pierro A. M. Tagnin M. Russo C. Fustos R. Corvetta D. (2016). Healing of a soft tissue wound of the neck and jaw osteoradionecrosis using platelet gel. Regen. Med. 11, 459–463. 10.2217/rme-2016-0031 27346565

[B50] Pietramaggiori G. Scherer S. S. Mathews J. C. Alperovich M. Yang H. J. Neuwalder J. (2008). Healing modulation induced by freeze-dried platelet-rich plasma and micronized allogenic dermis in a diabetic wound model. Wound Repair Regen. 16 (2), 218–225. 10.1111/j.1524-475x.2008.00362.x 18318807

[B51] Qu W. Wang Z. Hunt C. Morrow A. S. Urtecho M. Amin M. (2021). The effectiveness and safety of platelet-rich plasma for chronic wounds: A systematic review and meta-analysis. Mayo Clin. Proc. 96 (9), 2407–2417. 10.1016/j.mayocp.2021.01.030 34226023

[B52] Rajabi H. Sheikhani Shahin H. Norouzian M. Mehrabani D. Dehghani Nazhvani S. (2015). The healing effects of aquatic activities and allogenic injection of platelet-rich plasma (PRP) on injuries of achilles tendon in experimental rat. World J. Plast. Surg. 4 (1), 66–73. 25606479 PMC4298867

[B53] Rui S. Yuan Y. Du C. Song P. Chen Y. Wang H. (2021). Comparison and investigation of exosomes derived from platelet-rich plasma activated by different agonists. Cell. Transpl. 30, 096368972110178. 10.1177/09636897211017833 PMC8138303 34006140

[B54] Saputro I. D. Rizaliyana S. Noverta D. A. (2022). The effect of allogenic freeze-dried platelet-rich plasma in increasing the number of fibroblasts and neovascularization in wound healing. Ann. Med. Surg. (Lond). 73, 103217. 10.1016/j.amsu.2021.103217 35079361 PMC8767283

[B55] Scevola S. Nicoletti G. Brenta F. Isernia P. Maestri M. Faga A. (2010). Allogenic platelet gel in the treatment of pressure sores: A pilot study. Int. Wound J. 7 (3), 184–190. 10.1111/j.1742-481x.2010.00671.x 20455960 PMC7951528

[B56] Semenič D. Cirman T. Rožman P. Smrke D. M. (2018). Regeneration of chronic wounds with allogeneic platelet gel versus hydrogel treatment: A prospective study. Acta Clin. Croat. 57 (3), 434–442. 10.20471/acc.2018.57.03.05 31168175 PMC6536287

[B57] Semple J. W. Italiano J. E. Jr Freedman J. (2011). Platelets and the immune continuum. Nat. Rev. Immunol. 11 (4), 264–274. 10.1038/nri2956 21436837

[B58] Sethi D. Martin K. E. Shrotriya S. Brown B. L. (2021). Systematic literature review evaluating evidence and mechanisms of action for platelet-rich plasma as an antibacterial agent. J. Cardiothorac. Surg. 16 (1), 277. 10.1186/s13019-021-01652-2 34583720 PMC8480088

[B59] Simon L. M. Edelstein L. C. Nagalla S. Woodley A. B. Chen E. S. Kong X. (2014). Human platelet microRNA-mRNA networks associated with age and gender revealed by integrated plateletomics. Blood 123 (16), e37–e45. 10.1182/blood-2013-12-544692 24523238 PMC3990915

[B60] Smrke D. Gubina B. Domanović D. Rozman P. (2007). Allogeneic platelet gel with autologous cancellous bone graft for the treatment of a large bone defect. Eur. Surg. Res. 39, 170–174. 10.1159/000100490 17341879

[B61] Sun Y. Ma L. Ji M. Wang Z. (2022). Evidence map of recommendations on diabetic foot ulcers care：A systematic review of 22 guidelinesA systematic review of 22 guidelines. J. Tissue Viability 31 (2), 294–301. 10.1016/j.jtv.2022.03.001 35382991

[B62] Tae-ho C. Dae-seung B. Namjung K. Park J. h. Park C. (2015). Topical allogeneic platelet-rich plasma treatment for a massive cutaneous lesion induced by disseminated intravascular coagulation in a toy breed dog. Ir. Vet. J. 68, 4. 10.1186/s13620-015-0032-7 25763181 PMC4355006

[B63] Tamagawa-Mineoka R. (2015). Important roles of platelets as immune cells in the skin. J. Dermatol. Sci. 77 (2), 93–101. 10.1016/j.jdermsci.2014.10.003 25459165

[B64] Tashnizi M. A. Alamdari D. H. Khayami M. E. Moeinipour A. Amouzeshi A. Seifalian A. M. (2013). Treatment of non-healing sternum wound after open-heart surgery with allogenic platelet-rich plasma and fibrin glue-preliminary outcomes. Indian J. Plast. Surg. 46 (3), 538–542. 10.4103/0970-0358.122011 24459346 PMC3897101

[B65] Tian J. Lei X. X. Xuan L. Tang J. B. Cheng B. (2019). The effects of aging, diabetes mellitus, and antiplatelet drugs on growth factors and anti-aging proteins in platelet-rich plasma. Platelets 30 (6), 773–792. 10.1080/09537104.2018.1514110 30252623

[B66] Tyndall W. A. Beam H. A. Zarro C. O???Connor J. P. Lin S. S. (2003). Decreased platelet-derived growth factor expression during fracture healing in diabetic animals. Clin. Orthop. Relat. Res. 408, 319–330. 10.1097/00003086-200303000-00043 12616077

[B67] van der Bijl I. Vlig M. Middelkoop E. de Korte D. (2019). Allogeneic platelet-rich plasma (PRP) is superior to platelets or plasma alone in stimulating fibroblast proliferation and migration, angiogenesis, and chemotaxis as relevant processes for wound healing. Transfusion 59 (11), 3492–3500. 10.1111/trf.15535 31568583

[B68] Villela D. L. Santos V. L. (2010). Evidence on the use of platelet-rich plasma for diabetic ulcer: A systematic review. Growth factors. 28 (2), 111–116. 10.3109/08977190903468185 20001406

[B69] Vinod E. Vinod Francis D. Manickam Amirtham S. Sathishkumar S. Boopalan P. R. J. V. C. (2019). Allogeneic platelet rich plasma serves as a scaffold for articular cartilage derived chondroprogenitors. Tissue Cell. 56, 107–113. 10.1016/j.tice.2018.12.006 30736898

[B70] Wang A. Xu Z. Mu Y. Ji L. (2014). Clinical characteristics and medical costs in patients with diabetic amputation and nondiabetic patients with nonacute amputation in central urban hospitals in China. Int. J. Low. Extrem. Wounds 13 (1), 17–21. 10.1177/1534734614521235 24659623

[B71] Xie P. Deng B. Zhang X. Li Y. Du C. Rui S. (2022). Time in range in relation to amputation and all-cause mortality in hospitalised patients with diabetic foot ulcers. Diabetes. Metab. Res. Rev. 38 (2), e3498. 10.1002/dmrr.3498 34587332

[B72] Xu Y. Han K. Zhou Y. Wu J. Xie X. Xiang W. (2022). Classification of diabetic foot ulcers using class knowledge banks. Front. Bioeng. Biotechnol. 9, 81102. 10.3389/fbioe.2021.811028 PMC8918844 35295708

[B73] Xu Z. Ran X. (2016). Diabetic foot care in China: Challenges and strategy. Lancet Diabetes Endocrinol. 4 (4), 297–298. 10.1016/s2213-8587(16)00051-6 27016321

[B74] Ye F. Li H. Qiao G. Chen F. Tao H. Ji A. (2012). Platelet-rich plasma gel in combination with Schwann cells for repair of sciatic nerve injury. Neural Regen. Res. 7, 2286–2292. 10.3969/j.issn.1673-5374.2012.29.007 25538751 PMC4268730

[B75] Yotsu R. R. Hagiwara S. Okochi H. Tamaki T. (2015). Case series of patients with chronic foot ulcers treated with autologous platelet-rich plasma. J. Dermatol. 42, 288–295. 10.1111/1346-8138.12777 25615024

[B76] Zhang P. Lu J. Jing Y. Tang S. Zhu D. Bi Y. (2017). Global epidemiology of diabetic foot ulceration: A systematic review and meta-analysis. Ann. Med. 49 (2), 106–116. 10.1080/07853890.2016.1231932 27585063

[B77] Zhang X. Wang H. Du C. Fan X. Cui L. Chen H. (2022). Custom-molded offloading footwear effectively prevents recurrence and amputation, and lowers mortality rates in high-risk diabetic foot patients: A multicenter, prospective observational study. Diabetes Metab. Syndr. Obes. 15, 103–109. 10.2147/dmso.s341364 35046681 PMC8759996

[B78] Zhang Z. Y. Huang A. W. Fan J. J. Wei K. Jin D. Chen B. (2013). The potential use of allogeneic platelet-rich plasma for large bone defect treatment: Immunogenicity and defect healing efficacy. Cell. Transpl. 22 (1), 175–187. 10.3727/096368912x653183 22863146

[B79] Zhao Q. Ma Y. Lu Y. Chai Y. Zhou Y. (2019). Successful treatment of chronic lower extremity ulcers with allogeneic platelet-rich plasma and artificial dermis: A case report. Adv. Skin. Wound Care 32 (12), 550–552. 10.1097/01.asw.0000604176.47082.60 31764145 PMC7328864

